# CD44: a cancer stem cell marker and therapeutic target in leukemia treatment

**DOI:** 10.3389/fimmu.2024.1354992

**Published:** 2024-04-26

**Authors:** Shuang Wu, Yicheng Tan, Fanfan Li, Yixiang Han, Shenghui Zhang, Xiaofei Lin

**Affiliations:** ^1^ Laboratory Animal Center, the First Affiliated Hospital of Wenzhou Medical University, Wenzhou, Zhejiang, China; ^2^ Institute of Hematology, Wenzhou Medical University, Wenzhou, Zhejiang, China; ^3^ Wenzhou Key laboratory of Hematology, Wenzhou, Zhejiang, China; ^4^ Department of Hematology, The First Affiliated Hospital of Wenzhou Medical University, Wenzhou, Zhejiang, China; ^5^ Central Laboratory, The First Affiliated Hospital of Wenzhou Medical University, Wenzhou, Zhejiang, China

**Keywords:** CD44, leukemia, adhesion, migration, homing, differentiation, biomarkers, clinical application

## Abstract

CD44 is a ubiquitous leukocyte adhesion molecule involved in cell-cell interaction, cell adhesion, migration, homing and differentiation. CD44 can mediate the interaction between leukemic stem cells and the surrounding extracellular matrix, thereby inducing a cascade of signaling pathways to regulate their various behaviors. In this review, we focus on the impact of CD44s/CD44v as biomarkers in leukemia development and discuss the current research and prospects for CD44-related interventions in clinical application.

## 1 CD44 introduction

### 1.1 Structural basis of CD44

**Figure 1:**
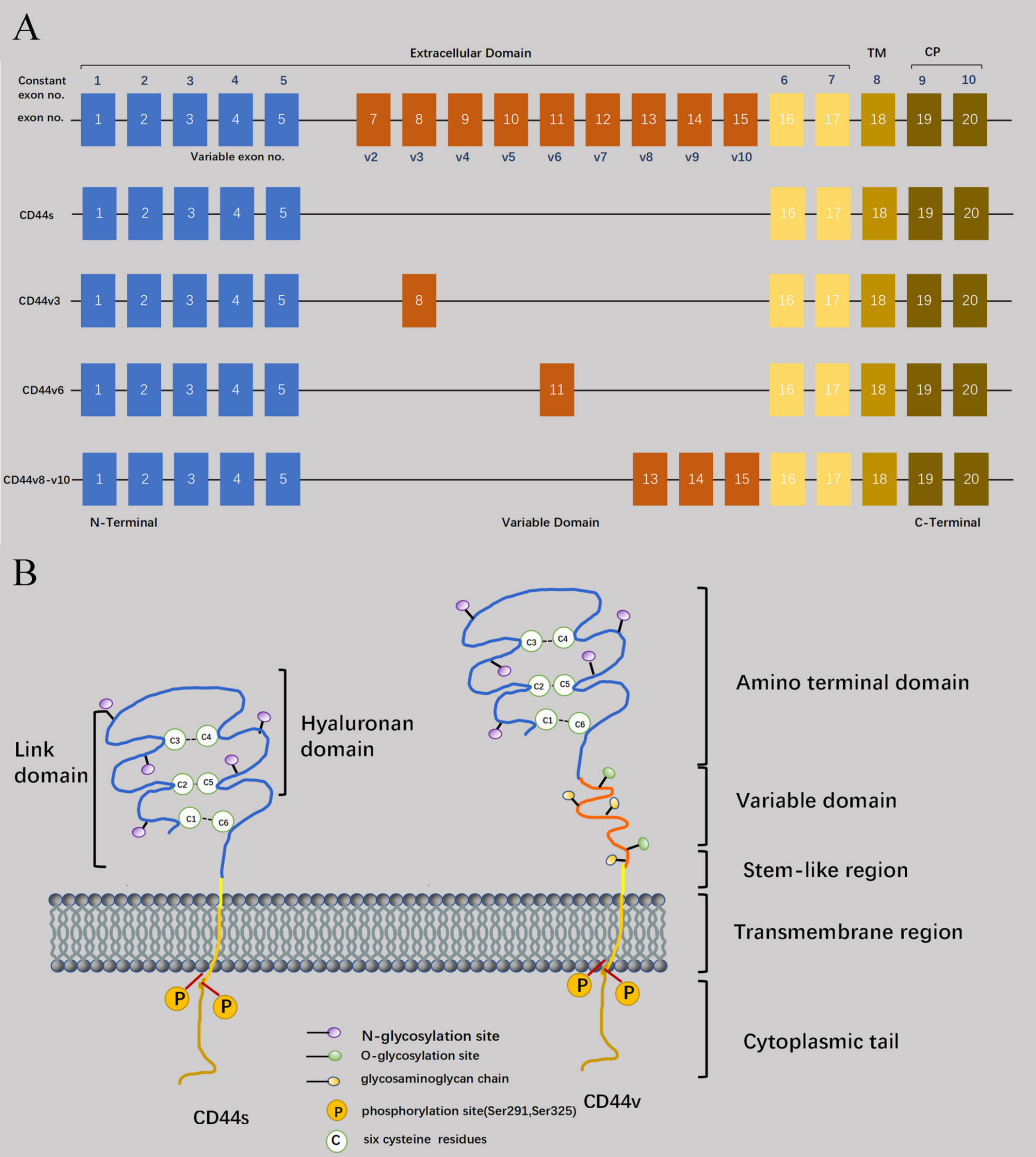
Structural basis of CD44. **(A)** CD44 is encoded by 20 exons in mice but 19 exons in humans. Exon 6 coding for CD44 variant 1 (CD44v1) is lacking in humans. Except for orange color exons, the rest of exons always expressed as a standard form of CD44 (CD44s), and up to ten exon variants can be inserted by alternative splicing. Full-length CD44, CD44s, CD44v3, CD44v6, and CD44v8-10 are shown schematically. **(B)** Four domains of CD44 glycoprotein are six cysteine residues (located at the amino acid termini), a stem-like region, transmembrane domain, and cytoplasmic tail. And the variable domain is between six cysteine residues and stem -like region.

CD44 is not just a single molecule localized on chromosome 11, but a transmembrane glycoprotein with molecular diversity produced by alternative splicing of multiple exons of a single gene and different post-translational modifications in different cell types (1). Exons located at both ends (1-5 and 16-20) are constitutive exons shared by all members of the CD44 family. In humans, the selective combination of various middle 9 exons during transcription give rise to different isoforms of CD44 precursors, thereby designating these middle 9 exons as variant exons (**Figure 1A**) (2). In addition to transcriptional shearing to generate different isoforms, CD44 also undergoes post-translational modifications. The most prevalent forms of glycosylation include N-linked and O-linked glycosylation (3). These post-translational modifications of CD44 isoforms have led to a more diverse ability to regulate cellular activities. For instance, following post-translational modification, different CD44 variants (CD44v) exhibit distinct modes of binding with its ligand Hyaluronic acid (HA) (4).

Regarding the molecular size, CD44 consists of 363 amino acids (37 kDa). However, due to glycosylation modifications and coding of mutant exons in the CD44 molecule, its actual molecular weight exceeds 37kDa and has been measured to be around 80-90 kDa (5). The extracellular region, transmembrane region, and short intracellular/cytoplasmic region at the C-terminus comprise the key characteristics of CD44 protein (**Figure 1B**) (6). Specifically, the extracellular domain can be subdivided into a globular structural domain consisting of six cysteine residues and a stem-like region in the extracellular near-membrane portion of the cell (7). As for the transmembrane and intracellular regions, both of them exhibit remarkable conservation in terms of their structure. The transmembrane region facilitates the oligomerization of CD44 at the cell membrane, thereby contributing to its localization in glycolipid-rich micromembrane compartments, as well as serving as a structural basis for interaction with lipid rafts (8).

### 1.2 CD44 receptor-ligand interaction

The biological characteristics initiated by CD44 are based on its ligand binding specificity and affinity. Commonly utilized ligands include HA, osteopontin (OPN), chondroitin, filaggrin/sulfated proteoglycans, fibronectin, and other compounds (9). Among these ligands, HA and OPN serve as the primary triggers for the majority of CD44-mediated activities.

### 1.3 Hyaluronic acid

CD44 interacts with HA, a common component of the extracellular matrix (ECM), in two ways (10). Firstly, cell membrane localized CD44 anchors soluble HA to the cell membrane through specific interactions. Secondly, immune or cancer cells expressing CD44 interacts with other cell membrane-expressed or anchored HA to mediate cell adhesion. The CD44 exists in three different states: (1) the resting state, where CD44 is unable to bind to its ligand HA; (2) the inducible and activated state, wherein CD44 requires specific activation by an activator to interact with HA; (3) the activated state, which does not necessitate an activator for interaction with HA (4). The majority of CD44 molecules present on the surface of hematopoietic cells are found in an inducible and activated state. Microstructurally, CD44 interacts with the ligand HA in a 160 amino acid fragment. In the specific subunit CD44 HA-binding domain (HABD) of CD44-bound HA and its corresponding amino acid sequence (The redox environment in cells affects the binding of HA through the disulfide bond formed by Cys77 and Cys97 in CD44), HA binds to CD44 primarily through hydrogen bonding and van der Waals forces rather than electrostatic and aromatic stacking interactions (11). The existence of two primary conformations, A and B, has been established for HABD (12). Notably, the B conformation exhibits a higher affinity compared to the other conformation. These distinct mechanisms can elucidate the varying degrees of conformational effects observed in CD44-HABD. Specifically, upon HA-binding stigmata, a C-terminal fragment undergoes a transition from an ordered state to a disordered state, consequently enhancing flexibility. The other observation is that HA binding causes an orientation change in the loop region of HABD near the arginine site at R41, consequently altering its affinity for HA. In addition, the affinity of CD44 for HA is influenced by the molecular weight of HA (13). Within a specific range, the binding capacity of CD44 to high molecular weight HA surpasses its interaction with low molecular weight HA, potentially due to an abundance of binding sites between high molecular weight HA and CD44. However, the binding affinity between these two molecules decreases as the molecular weight of HA increases, rather than intensifying (13). In addition, numerous other factors can influence the binding ability of CD44 to HA. For instance, the extent of glycosylation in the extracellular domain of CD44 and phosphorylation of specific serine residues at the cytoplasmic end regulates its HA-binding capacity. The regulation also involves cytokines and matrix metalloproteins. Furthermore, TNF-α can also induce HA binding to CD44 on peripheral blood monocytes.

### 1.4 Osteopontin

The primary roles of OPN encompass serving as an integral constituent of the extracellular matrix and functioning as a cytokine (14). In the C-terminal sequence of OPN, there is also a non-RGD cell adhesion site segment, and OPN acts as a signaling molecule by binding to CD44 on the cell surface in an RGD-independent manner (15). Phosphorylation is an important self-regulatory mechanism for OPN; phosphorylated OPN specifically interacts with cell surface integrin receptors, while the dephosphorylated form selectively binds to the CD44 receptor and elicits distinct functional outcomes (16). The accessibility of the extracellular domain to the compressed core region of OPN is also a pivotal determinant for effective interaction with CD44. Furthermore, glycosylation of OPN may prevent its interaction with CD44. In addition to the extensive diversity of OPN and CD44 proteins resulting from shearing, glycosylation, phosphorylation, etc., their potential co-interacting partners such as heparin, hyaluronan and integrin αVβ3 also give rise to numerous interaction possibilities. Specifically, research evidence suggests that heparin binding on OPN plays a paramount role in its interaction with CD44 (17), which facilitates its interaction with homologous receptors, such as variant exon 3 on CD44 (CD44v3), thereby forming a molecular bridge. The distinct combinations need further investigation to understand how they produce different signals on receptor cells.

## 2 CD44 related molecular mechanisms

### 2.1 Molecular mechanisms involved in migration

**Figure 2:**
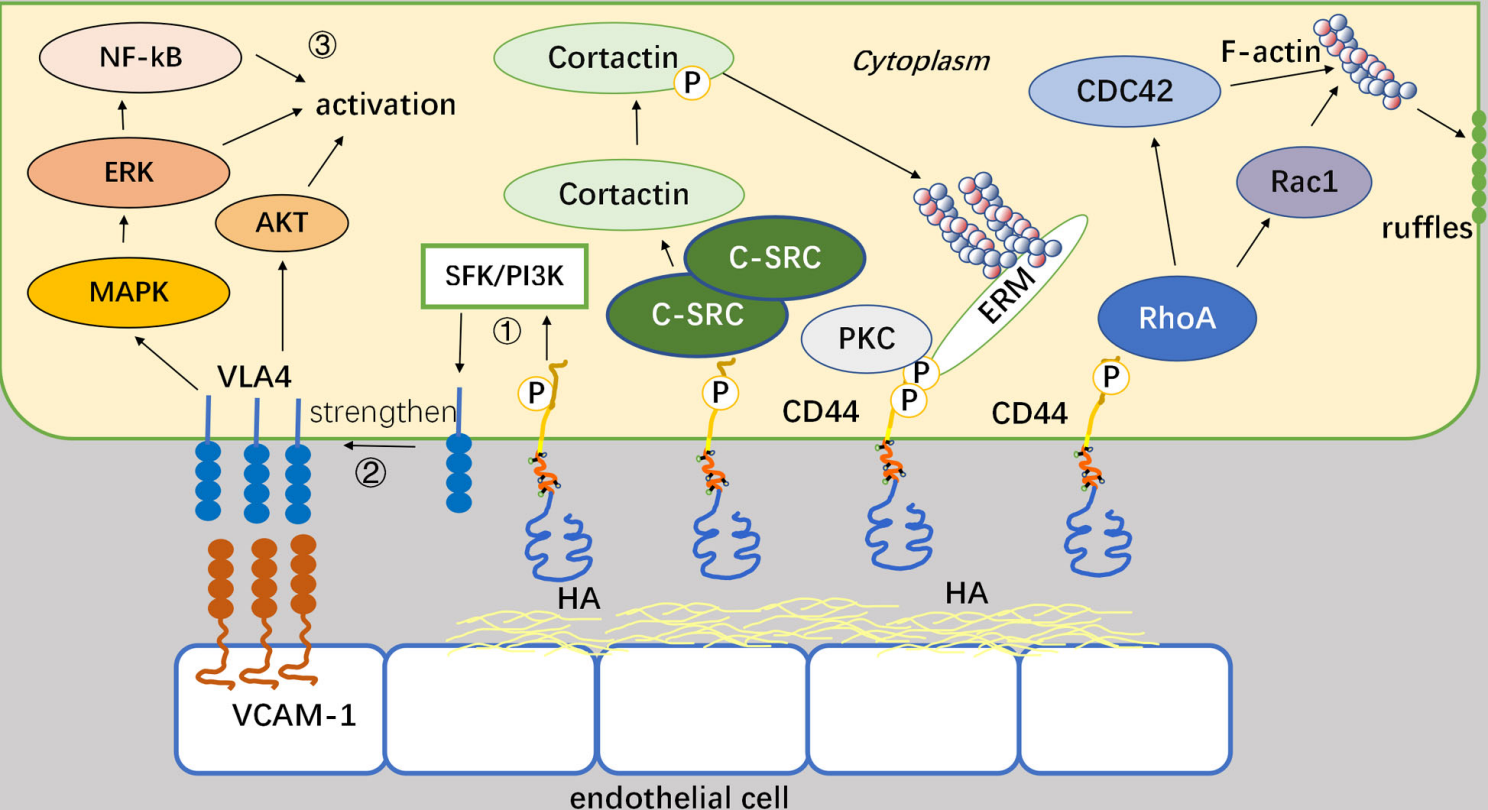
Mechanisms by which CD44 regulates the motility and adhesion. Binding of CD44 to HA can affect the intracellular system of backbone proteins and thus mediate cellular movement. CD44 connects to actin via the ERM as a bridge, and this process requires PKC activation as a prerequisite. It also recruits and activates c-Src, and regulates cell migration capacity by phosphorylating the actin Cortactin cytoskeletal protein tyrosine. After activation of Rho GTPase, two downstream factors, CDC42, and Rac1, regulate the cytoskeleton and cell membrane fold structure, respectively, thereby altering cell motility.

CD44 is involved in the regulation of cell motility through intracellular structural domains, which constitutes a pivotal mechanism. There are three prevailing mechanisms involved: firstly, phosphorylation of the intracellular terminus of CD44 occurs following its interaction with HA and subsequent activation by protein kinase c (PKC) (18), and then ezrin, radixin, moesin (ERM) proteins serves as a link connecting CD44 and filamentous actin (F-actin) and ultimately influence cellular motility (19). Secondly, c-Src kinase is recruited to the CD44 site and activated through CD44-HA interaction (20). Activated c-Src increases tyrosine phosphorylation of a cytoskeletal protein called Cortactin (21). Consequently, its ability to cross-link F-actin decreases, which regulates cell migration capacity and promotes cell recruitment. Lastly, RhoGTPase triggers two different signaling pathways: firstly, it regulates F-actin via CDC42, thereby modulating the cytoskeleton and facilitating cell recruitment (22, 23). Secondly, it activates Rac1 to modulate the fold structure of the cell membrane in order to regulate cells (24). For example, CD44v3 has been discovered to promote Rac1 signaling by interacting with Tiam1, resulting in the cytoskeleton-mediated breast tumor cell migration (25). CD44-HA binding induces the aggregation of CD44 at the leading edge of cells, where it interacts with the actin cytoskeleton and its cytoplasmic tail (26). Activation of Rac1 through RhoA signaling has been proven to promote a shift towards a migratory phenotype once cells have attached to the endothelium. Rac1 activation plays a pivotal role in CD44-mediated cytoskeletal reorganization. When CD44 binds to c-Src, activated c-Src kinases phosphorylate Vav (mostly Vav1 and Vav2, two guanine exchange factors regulated by Rho GTPases) and consequently increase Rac1 expression (27). Under the help of F-actin, CD44 translocates from the rear end to the leading edge in this process. HA-CD44 binding promotes the combination of RhoA-specific p115RhoGEF with CD44 and further activates the RhoA-ROK signaling pathway. Finally, ROK phosphorylates ankyrin and enhances F-actin linkage (**Figure 2**) (28).

CD44 can synergistically interact with VLA-4 integrins through intracellular signaling pathways, aside from its significant adhesion role (29). VLA-4 is a heterodimer formed by CD49d and CD29 (30). Moreover, as a key coordinator of the interaction between leukemia cells and the bone marrow microenvironment, CD44 plays an important role in mediating cellular homing. In general, VLA-4 often interacts with VCAM-1, and its thermosensitivity and affinity for the corresponding ligands often undergo alterations upon exposure to external stimuli (31). CD44 can act as a regulatory factor in the above process. Specifically speaking, CD44 enhances VLA-4 activity by inducing intracellular molecular pathways through binding to HA or other extracellular matrices, which in turn enhances the adhesion of leukemia cells to VCAM-1. VLA-4 interacts with VCAM-1 to promote Akt, MAPK, NF-κB, and mTOR signaling, while reduce apoptosis in acute myeloid leukemia (AML) cells. Overall, CD44 modulates the affinity of VLA-4 in AML cells for VCAM-1 in peripheral blood and retains AML cells in the bone marrow microenvironment. In contrast to integrin VLA-4, CD44 can interact with integrin α6β4 to form a complex, which activate intracellular cytoskeletal proteins and their signaling pathways, thereby modulating the c-Src and Ras signaling cascade and promoting tumor cell migration (32). Notably, CD44-α6β4 can be delivered to the target region through the paracrine action of exosomes. Moreover, hepatocytes used in the experiments can selectively uptake tumor exosomes with high expression of CD44. Ultimately, this process supports tumor metastasis by stimulating cytokine production, pro-inflammatory factors release, and growth factor secretion for pre-metastatic niche formation. Although the mechanism of action regarding CD44 exosomes on leukemia cells still needs further research, it undoubtedly provides a new approach for targeting CD44 on leukemia.

Lipid rafts are cholesterol- and sphingolipid-rich microstructural domains in the plasma membrane (33). CD44 is predominantly localized in lipid raft clusters. Co-localization of CD44 in specific membrane environments is essential for cell adhesion and migration. The aggregation of CD44 by lipid rafts occurs through two main pathways (34). One type of palmitoylation retains CD44 in the lipid raft structural domain and partially inhibits its interaction with intracellular signaling molecules. However, another lipid knows as phosphatidylinositol-4,5-bisphosphate (PIP2) can expedite the process of CD44-junction complex formation (35). Several factors have been identified to influence lipid rafts. For example, methyl-β-cyclodextrin can deplete cholesterol and reduce CD44 aggregation on lipid rafts, thereby enhancing CD44 binding to HA on T cells. In addition, high levels of cholesterol can promote the entry of CD44 into lipid rafts, resulting in decreased binding between CD44 and Ezrin and subsequently inhibiting the migration and invasion of tumor cells (36).

Paxillin, an adhesive patch adaptor protein, is mainly localized at focal adhesions (FAs) (37). Cell movement and migration require the accumulation of paxillin at nascent FAs at the leading edge to recruit adhesion complexes, including the tyrosine kinases FAK, and elimination of FAs at the rear edge (38). The engagement of CD44 involves promoting tyrosine phosphorylation and activation of FAK under the premise of combination with HA. In the acidic milieu, hyaluronidase-2 and cathepsin B are activated to support ECM degradation and activate ROK, and subsequently induce the phosphorylation of Na+/H+ exchanger 1 (39).

### 2.2 Mechanisms involved in growth and proliferation survival

**Figure 3:**
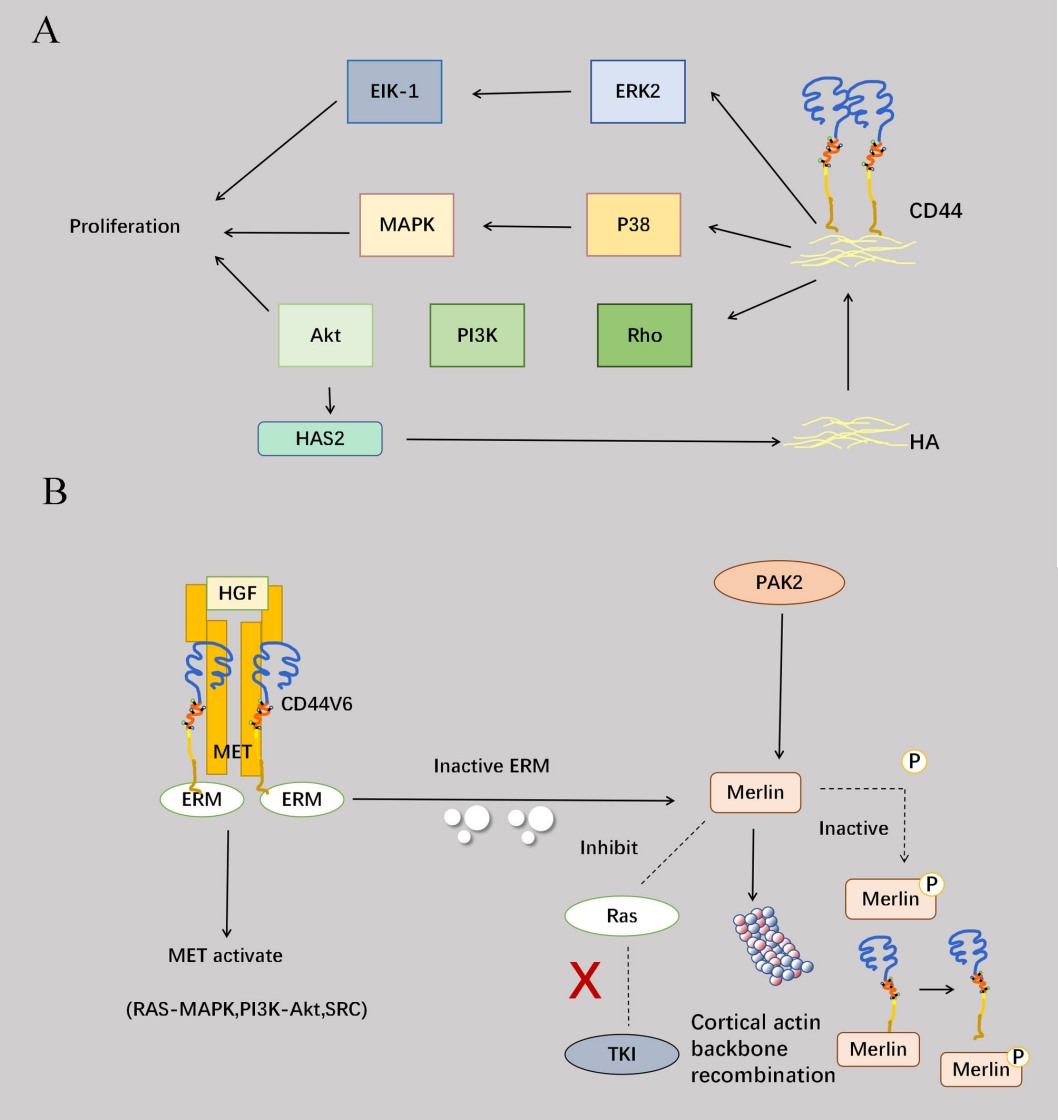
Involvement of CD44 in proliferation. **(A)** Binding of CD44 to HA can activate the PI3K-Akt pathway through RHO, and the activation of the Akt pathway can promote the increase of HAS2 synthase, which in turn can enhance the effect of CD44 and HA to form a positive cycle, overcoming apoptosis and maintaining cell survival. CD44 also binds to HA and activates the P38-MAPK pathway to enhance cell proliferation. Activation of the extracellular kinase ERK2 phosphorylates EIK-1 and ultimately promotes cell migration and proliferation. **(B)** CD44v6 forms a trimeric complex with Met and HGF and promotes Met activation. Furthermore, the interaction of the CD44 cytoplasmic tail with ERM proteins is required to activate the Ras-MAPK pathway. CD44v6-ECM binding also promotes PI3K/Akt pathway activation and Met transcription. Another protein, Merlin, competes with ERM at the intracellular end of CD44, and Merlin activation occurs after ERM protein inactivation. Merlin activation causes reorganization of the cortical actin cytoskeleton, prevents Ras activation and Ras-dependent signaling, and inhibits signaling by various receptor tyrosine kinases. The serine/threonine protein kinase PAK2 phosphorylates Merlin, but also leads to Merlin inactivation and inhibits its binding to CD44. The addition of high cell density or high molecular weight HA triggers the dephosphorylation of Merlin, leading to the formation of growth inhibitory complexes that limit cell proliferation. Therefore, ERM and Merlin proteins act as “switches” to control cell proliferation.

CD44 is capable of binding to ligands to activate a range of intracellular signaling pathways that regulate cellular growth, proliferation, survival and various other activities. Specifically, the interaction between CD44 and HA triggers the activation of Rho, which in turn stimulates the PI3K pathway and activates the downstream serine/threonine kinase Akt. Notably, HA establishes a positive feedback loop with Akt by inducing continuous activation of Akt signaling to counteract apoptosis and maintain cellular survival (40). CD44 also enhances cell proliferation by activating the p38 mitogen-activated protein (MAP) kinase (41). Furthermore, extracellular kinases can be involved in regulation, such as extracellular-associated kinases 1 and 2 (ERK1/2), which regulate the activation and proliferation of endothelial cells. Activated ERK2 can promote cell migration and proliferation by phosphorylating EIK-1 (23). CD44v6, as a tumor marker in the CD44 family, forms a trimeric complex with MET and HGF to promote MET activation and activate three downstream pathways: RAS-MAPK activation, PI3K/Akt promotion, and MET transcription enhancement (**Figure 3A**) (42). Among signaling molecules, ERM and Merlin are commonly recognized as pivotal regulators of cell proliferation, and Merlin and ERM engaging in competitive binding for the CD44 intracellular site (43). Upon activation, Merlin facilitates cytoskeletal reorganization while concurrently suppressing RAS activation and downstream tyrosine kinase signaling. Conversely, phosphorylation-induced inactivation of Merlin impedes its interaction with CD44 (**Figure 3B**) (44).

### 2.3 CD44 cleavage

**Figure 4:**
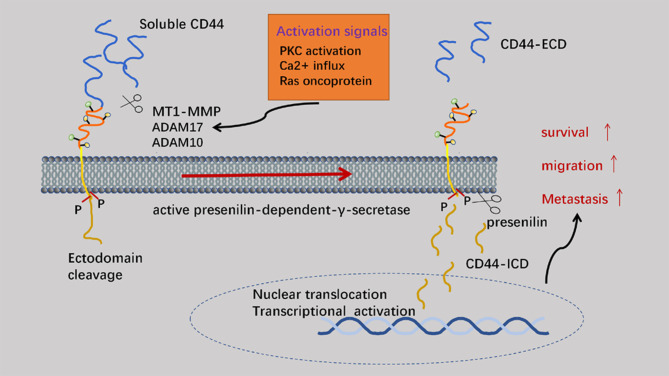
The cleavage of CD44. The ectodomain of CD44 is mainly cleaved by MMP (membrane-associated metalloproteases) to generate CD44 ECD. On the premise of previous process, CD44 ICD can be generated by intramembranous cleavage, which mediated by presenilin (PS)-dependent-γ-secretase. CD44 ICD can act as a signal transduction molecule to activate transcription.

Proteolytic cleavage of CD44 is a key regulatory event dependent on CD44 cell-matrix interactions and signaling pathways. The process of matrix metalloproteinase (MMP)-mediated shearing of the CD44 extracellular structural domain to cleavage of the intracellular structural domain to the final release of intracellular structural domain fragments (ICDs) to act as signaling transcription molecules is strictly sequential (45). After cleavage of the exo-structural domain, the presenilin-dependent-γ-secretase-dependent shearing within the membrane is initiated. Finally, the intramembrane cleavage product CD44-ICD can act as a signal transduction molecule that is translocated to the nucleus and activates transcription. Exo-structural domain cleavage of CD44 correlates with malignancy in human tumors (46). Extracellular soluble CD44 (sCD44) is a competitive inhibitor of endogenous HA-CD44 interaction (47). The main MMPs involved in exon cleavage are MT1-MMP, ADAM10 and ADAM17. Specifically, MT-1-MMP binds to CD44 through the PEX (C-terminal hemopexin-like domain) structural domain and localizes to the ciliary membrane (48). CD44 acts as a linker between MT1-MMP and the actin cytoskeleton in inactive cancer cells. Co-activation of the molecules PKC and RAC induces the aggregation of ADAM17 and CD44 at the leading edge of migrating cells, and accumulation of ADAM17 is activated and undergoes cleavage of the CD44 ecto-structural domains allowing the cells to crawl efficiently on the ECM, with the extended lamellae also inducing mechanical stretching of the cells (49). Extracellular calcium ions flow into the cell through stretch-activated calcium channels. With the accumulation of intracellular calcium ions, ADAM10 is rapidly activated to aid CD44 cleavage. CD44 cleavage promotes attachment of newly synthesized CD44 to the ECM. The release of sCD44 from the C-terminus triggers the intramembrane cleavage of CD44 ectodomain cleavage, a process dependent on presenilin-dependent-γ-secretase. Finally, CD44-ICD translocates to the nucleus and activates the transcription of various genes including CD44. The activation of CD44-associated proteases can facilitate the movement of cells (50). Therefore, profound understanding the underlying mechanisms of CD44 cleavage could develop new therapeutic approaches for cancer cell invasion and metastasis. Elevated levels of sCD44 in plasma are correlated with malignant diseases and immune activation. Additionally, competition between the ectodomain released by the receptor and cell surface CD44 can antagonize the effect of membrane-bound CD44, which might also shed light on CD44-related treatments (**Figure 4**) (51).

### 2.4 Molecular mechanisms involved in the metabolism of CD44

CD44 is capable of binding to copper and facilitating its internalization into the cellular interior (52). Cu(II) can exert influence on cellular metabolism and function through diverse pathways, such as involvement in macrophage activation and inflammatory responses. During macrophage activation, upregulation of CD44 leads to an elevation in Cu(II) levels. Additionally, Cu(II) also catalyzes the NAD(H) redox cycle, which promotes metabolic changes and subsequent epigenetic alterations leading to an inflammatory state. This mechanism has been more extensively studied in Wilson and Menkes disease, characterized by impaired copper transporters leading to potential copper accumulation and toxic effects, along with other chronic metabolic diseases (53), further verification is required regarding its precise mode of action on leukemia cells. Moreover, CD44 plays a crucial role in regulating adipogenesis and adipocyte function through PPAR-γ and cell cycle-related pathways. The elimination of CD44 improves adipose tissue inflammation and insulin resistance in obesity. CD44 also regulates glucose metabolism in various cancer cells (such as PC-3 cell line and small cell neuroendocrine carcinoma cells), while also modulating ROS levels and cellular proliferation within neoplastic cells (54). Finally, CD44 is implicated in the regulation of cellular autophagy by downregulating PIK3R4 and PIK3C3 levels through CD44-ICD and disrupting the STAT3-dependent PtdIns3k complex to exert negatively modulate on autophagy (55).

## 3 The role of CD44 in leukemia

### 3.1 Expression of CD44 and its variants in leukemia cells

Leukemia is a hematological disorder characterized by diminished levels of normal blood cells and impaired bone marrow function due to uncontrolled proliferation of leukemia cells within the bone marrow. The expression of CD44v is exclusively detected in leukemia cells, while being absent in bone marrow, peripheral blood, and CD34^+^ hematopoietic stem cells (HSCs). The complexity of CD44v expression in leukemia cells has been associated with low remission rates and high relapse rates during treatment, indicating an unfavorable prognosis. In addition, CD44v expression in leukemia cells is influenced by numerous factors, including inherent individual variations that are common and unavoidable, as well as the specific type of leukemia (e.g. the CD44v7 expression level is lower on lymphocytes than monocytes and granulocytes) (56). Additionally, some leukemia FAB subtypes, specifically AML M3 and M5 subtypes, exhibit elevated levels of CD44v expression in conjunction with genotype variations. For instance, individuals carrying the rs13347 TT and CT genotypes demonstrate higher CD44v expression level compared to those with the rs13347 CC genotype (57). The presence of various complex patterns of CD44 variant exons, particularly exon v6, has been observed in the majority of leukemia samples (58). The combination of CD44v6 and HA enhances cell resistance to apoptosis and activates the PI3K/Akt signaling pathway (42). Upregulation of CD44v6 can also elevate the expression of HA synthase gene. Due to its widespread presence and varying expression levels at each stage of leukemia development and across diverse subtypes of leukemia, CD44 has been considered as a valuable biomarker (**Table 1**). In the field of leukemia treatment, CD44 can also be applied for universal minimal residual disease (MRD) monitoring, as its high expression levels are always associated with poor prognosis (91). This phenomenon may be related to the reciprocal interaction between CD44 protein expression and oncogenes (92).

**Table 1:** The expression of CD44 subtypes and their clinical significance in leukemia.

Reference	type of leukemia	CD44 type	Clinical significance
(59)	ALL, AML, MDS	sCD44	prognosis, disease progression
(60)	ALL	CD44	differentiation, metastasis
(61)	ALL	CD44	prognosis
(62)	ALL	CD44	disease progression, homing
(63)	ALL	CD44, CD44 v6	prognosis, relapse
(64)	ALL	CD44	prognosis
(65)	ALL	CD44	MRD detection
(66)	B-precursor ALL	CD44	stages of B-precursor development
(67)	CLL	sCD44	prognosis
(68)	CLL	CD44 v3,4,5,6,7,9,10	prognosis
(69)	CLL	CD44	prognosis
(70)	CLL	CD44	aggressive forms, chemotaxis, autophagy
(71)	CLL	sCD44	disease progression
(72)	CLL	CD44	disease progression
(73)	AML, MM	CD44 v6	engraftment
(74)	AML	CD44	prognosis
(75)	AML	CD44 v6	survival, leukocyte activation, malignant transformation
(76)	AML	CD44	prognosis
(77)	AML	CD44 v6	prognosis
(78)	AML	CD44	disease progression
(79)	AML	CD44	relapse
(80)	AML	CD44	risk
(81)	AML	CD44	disease progression
(82)	AML	CD44	relapse
(83)	AML	CD44	adhesion, drug resistance
(84)	AML	CD44	survival
(85)	AML	CD44	disease progression
(86)	AML	CD44	adhesion, drug resistance
(87)	AML, ALL	CD44	homing, adhesion
(88)	refractory AML	CD44	survival
(89)	MDS	CD44	prognosis
(90)	CML	CD44	drug resistance

### 3.2 CD44 mediates the rolling of blood cells

Different types of selectins typically act at different stages of cell rolling as important molecules located in endothelial cells for interaction with CD44. For example, P-selectin is known to affect the fast roll of the prophase, while E-selectin mediates CD44 engagement during the slow rolling of neutrophils (93, 94). Furthermore, in various T cell conditions, CD44 collaborates with other ligands like HA and integrins to facilitate the rolling and adhesion of T cells. Moreover, CD44 plays a pivotal role in promoting the recruitment of neutrophils and macrophages to inflammatory sites.

### 3.3 CD44 correlates with chemoresistance

As an important prognostic indicator, the upregulation of CD44 exhibits statistically significant implications in the cell fate of many cancer cells, including leukemia cells. Moreover, it has been observed that drug-resistant cells tend to display elevated levels of CD44 expression (95). Functionally, CD44 interacts with the extracellular matrix, particularly HA, to activate Nanog through some key signaling pathways, such as the Wnt/β-catenin, and PI3K/Akt (96), the targeted Nanog has been utilized as a mediator to enhance the expression of drug resistance genes. CD44v6 is considered a premetastatic niche of tumor cells and correlates with drug resistance (97). CD44v3 has been implicated in the regulation of chemotherapy resistance through the Oct-Sox2-Nanog signaling pathway (98). It is noteworthy that HA of different sizes has diverse effects on CD44-mediated drug resistance. Low molecular weight HA binding to CD44 tends to induce the internalization of MDR, while high molecular weight HA binding to CD44 promotes the expression of MDR on cell membrane (99).

### 3.4 CD44 is involved in proliferation and anti-apoptosis of leukemia cells

The prognosis is inversely correlated with the expression levels of CD44 in leukemia cells and their rate of increase; however, further evidence is needed to fully support the underlying mechanism. It has been discovered that CD44 promotes the proliferation of leukemia cells through upregulating expression of anti-apoptotic protein myeloid cell leukaemia-1 (MCL-1) (100). Chang et al. found that the downregulation of CD44 led to a decrease in β-catenin expression, thereby inducing leukemia cell cycle to arrest in the G0/G1 phase, where gene transcription is blocked, and cell proliferation is inhibited (101). Consequently, it can be inferred that CD44 exerts regulatory control over leukemia cells through the Wnt/β-catenin pathway.

## 4 CD44-related measures applied in clinical treatment

**Figure 5:**
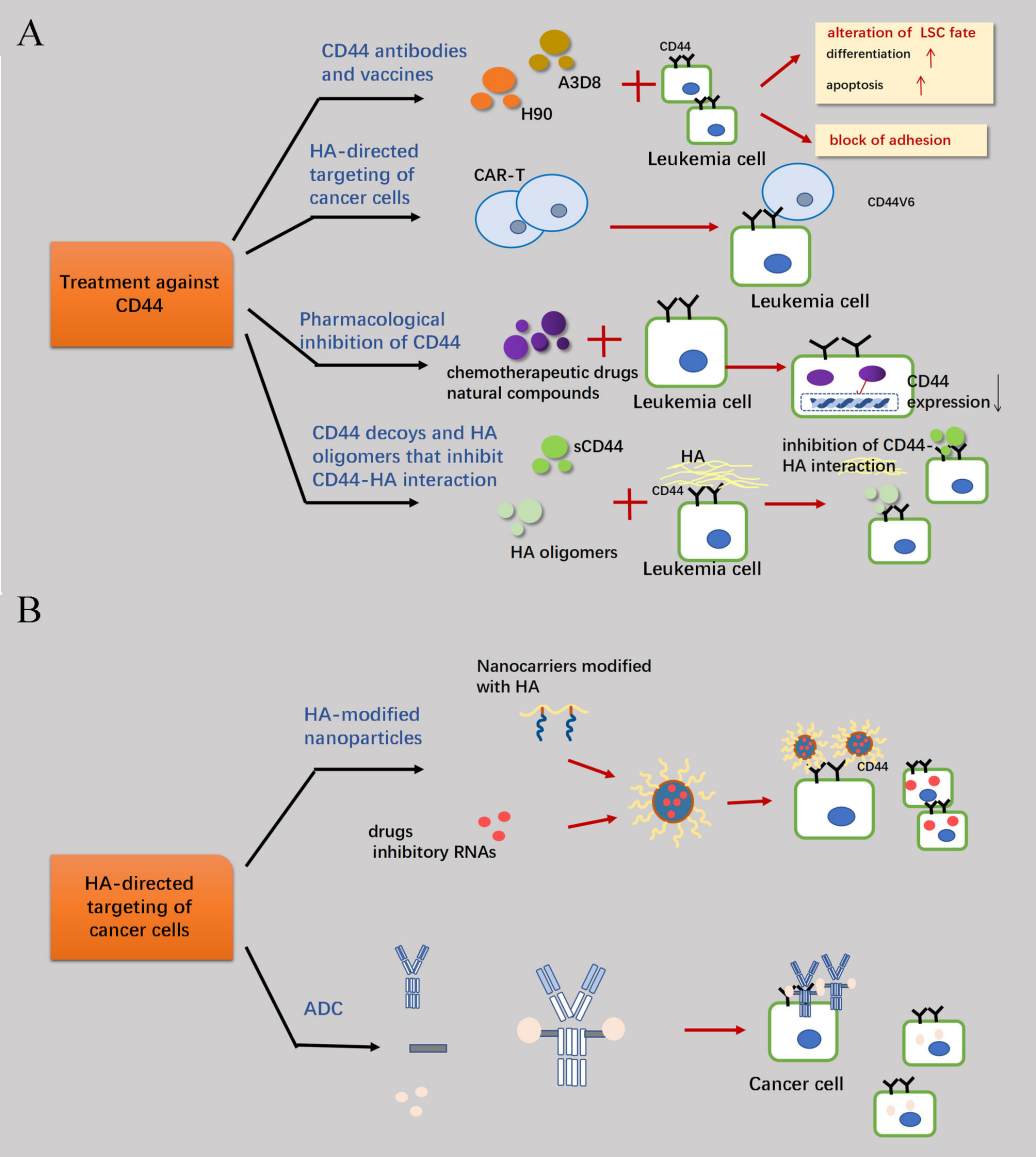
Therapeutic measures concerning CD44. **(A)** Because of the high expression of CD44 molecules in target cells and their involvement in mechanisms such as anti-apoptosis of tumor cell proliferation, blocking the signaling pathways of relevant cellular activities by combating CD44 is a feasible therapeutic strategy. Primary treatments are the development of CD44-targeted drugs, immune-representative therapies such as the elimination of AML cells through CAR-T cells which associated with CD44v6 and the enhancement of therapeutic efficacy through the screening of drugs that can reduce the transcriptional expression of CD44 in combination with conventional chemotherapeutic agents. Finally, the binding of CD44 to HA can be interfered with by sCD44 and HA oligomers. **(B)** The high affinity between CD44 and HA can also be an important channel for drug delivery into the cell. Through the HA linkage, CD44 antibodies can be combined with drugs to form antibody-drug conjugates (ADCs), which can more efficiently target and attack highly CD44-expressing tumor cells.

CD44 is frequently overexpressed in the tumor stroma across various malignancies, and both neoplastic cells associated CD44 and HA within the stromal microenvironment have been targeted for anti-cancer therapy. Current perspectives on the treatment of CD44 can be broadly categorized into two domains. One approach focuses on directly targeting CD44 itself (**Figure 5A**), considering its involved in the self-regulatory activities of tumor cells such as proliferation, anti-apoptosis, drug resistance and other mechanisms. Targeting CD44 hinders relevant signaling pathways associated with these activities. Moreover, as an adhesion molecule, CD44 is involved in cell adhesion and chronic rolling to retain tumor cells in the appropriate microenvironment. By blocking CD44 binding to ligands, it facilitates tumor cells evading the tumor microenvironment for better exposure to drugs and immune cellular strikes - particularly crucial for eliminating residual leukemia stem cells (LSCs) after HSC transplantation to reduce postoperative recurrence (1). The other category involves the utilization of CD44 as a more accurate drug delivery channel and tumor cell localizer, considering its robust affinity for HA and the properties of the HA molecule itself (102). Even in passive immunotherapies including CAR-T cell therapy, CD44v6 is considered a viable T-cell localization molecule due to its high expressivity in AML cells. Specifically, for the first approach to CD44 itself, it is common to develop monoclonal antibodies about CD44 or chemotherapeutic agents that inhibit the binding of CD44 to HA by utilizing sCD44 and specific molecular sizes of HA as inhibitors. Alternatively, drug delivery via CD44-HA has gained significant attention in recent years, particularly regarding the utilization of HA as a special material for nanoparticle modification and its application as an intermediary bridge facilitating communication between the drug and CD44-overexpressing tumor cells (**Figure 5B**).

### 4.1 CD44-targeted drugs

Depending on their chemical structure, CD44-targeted drugs can be classified as monoclonal antibodies (mAbs) or small molecule compounds. The overexpression of CD44 variant isoforms on the surface of tumor cells, coupled with the ability of cellular overexpression of CD44 to increase the secretion of cytosolic HA and create a favorable microenvironment for the tumor cells, positions CD44 as a promising target molecule. CD44 antibodies have two distinct mechanisms of action: alteration of LSC fate, including inhibition of LSC differentiation blockade and disruption of LSC adhesion. The anti-CD44 mAbs H90 and A3D8 not only inhibit the proliferation and self-renewal of LSCs, but also promote leukemia cell differentiation and induce apoptosis. A3D8 mAb induces apoptosis via the engagement of the serine protease-regulated pathway (103). Notably, despite these two antibodies share a common target protein, they exhibit distinct mechanisms of action (104). Furthermore, the combination of A3D8 and H90 effectively induces terminal differentiation of leukemic blasts in AML-M1 to AML-M5 subtypes, which are the most prevalent variants (105). A3D8 inhibited the proliferation of HL-60 cells, and its differentiation mechanism was associated with the up-regulation of p21 cip1 expression and downregulation of cyclin D1 and CDK4 expression (106). The proliferation inhibitory effect of A3D8 on HL-60 cells related to the downregulation of amino acid terminal kinase expression. Another familiar antibody called RG7356 (a recombinant anti-CD44 IgG1 humanized mAb) elicits caspase-dependent apoptosis in leukemic cells (107). In most practical clinical applications, antibodies are utilized in combination with drugs to potentiate their effects. The combined treatment with cyclopamine (a plant-derived steroidal alkaloid) and CD44 antibody effectively enhances the therapeutic effects of inducing the differentiation of leukemia by hindering the Hedgehog signaling pathway (108). The addition of anti-CD44 mAbs provides benefit to “ATRA-FICZ” (FICZ: 6-Formylindolo (3, 2-b) carbazole) enhances ATRA-induced differentiation therapy, especially in APL patients (109). Nonetheless, careful consideration should be given to the sequential administration of antibodies and chemotherapeutics. For example, A3D8 mAb entirely eliminates the ceramide, a lipid second messenger participating in the apoptotic signaling pathway triggered by daunorubicin. Therefore, administering anti-CD44 mAbs after apoptosis-inducing drugs can avoid interfering with their original effects.

Undoubtedly, the development of targeted drugs encounters numerous challenges. The meticulous identification and validation of novel drug targets and their precursors, along with the arduous process of developing targeted drugs with extensive research and development cycles, have contributed to the issue of exorbitant pricing. Furthermore, while targeted drugs are exhibiting efficacy against tumors with high specific expression of certain oncogenes, they demonstrate limited effectiveness against other tumor types. Although the precise mechanisms underlying drug resistance remain incompletely elucidated, it is widely acknowledged that mutations in genes or prolonged drug exposure may cause drug resistance; additionally, epigenetic or cytokine abnormalities might also induce such resistance (110). Furthermore, targeting CD44 will inevitably have some cytotoxicity due to its expression on immune cells, such as NK cells, and CD8^+^T cells (111). A commonly employed approach to address the aforementioned challenges is protein-targeted chimerism (PROTAC) (112), a method that can transform most non-druggable protein targets into druggable ones. In addition to PROTAC, subsequent advancements have been made in autophagosome-targeted complexes using lysosome-targeted technology and autophagosome-targeted degradation by autophagosome-bound compounds since 2019 (113). In addition, progress has been made in the development of dual-targeted or multi-targeted drugs. Malignant tumors are intricate network regulated by multiple factors with diverse etiologies, merely intervening in one target or inhibiting a pathway following the strategy of a single-target drug triggers activation of another related pathway, leading to suboptimal efficacy of the single target and rapid development of resistance. Therefore, apart from solely targeting CD44, attempts should be made to simultaneously target another highly characterized molecule overexpressed in tumor cells to mitigate the rate of resistance and improve targeting precision. Given the vast number of compounds and target molecules, relying solely on traditional manual screening techniques to ensure efficiency and accuracy becomes challenging. Therefore, leveraging artificial intelligence and big data technologies have proven to be beneficial in this regard. Secondly, regarding the issue of CD44 toxicity, it has been previously mentioned in section 3.1 that distinct subtypes of CD44 are highly expressed by different leukocyte species and different leukemia subtypes. Although NK cells possess the capability to exhibit elevated levels of CD44 expression, and it has been demonstrated that signaling through CD44 enhances cytotoxicity in NK cells. Additionally, expression of CD44-positive liver-resident CD8^+^ T cells was also found to be critical for immune clearance of hepatitis B virus. However, resting NK cells constitutively express inactive forms of CD44 which do not bind to HA. In addition, pro-inflammatory cytokines play an important role in activating CD44 on NK cells. Although stimulation of resting NK cells with interleukin-12 (IL-12) or IL-18 resulted in increased CD44 expression, only IL-2 or IL-15 led to upregulation and activation of CD44 (111). Cytokine-induced upregulation and activation of CD44 is not associated with NK cell proliferation. That is, HA alone exerts minimal impact on IFN-γ production, whereas low molecular weight HA effectively enhances of IFN-γ production when combined with IL-2, IL-12, or IL-18. Exploiting the unique properties of HA and its association with CD44 holds promise for modifying NK cells and subsequently activating their recognition mechanism to expedite solid tumor treatment, offering a prospective avenue for NK cell-mediated immunotherapy.

### 4.2 Pharmacological inhibition of CD44

Except for directly targeting the interaction of HA and CD44, several natural compounds or chemotherapeutic drugs have been discovered to indirectly inhibit LSCs through reducing CD44 expression. ATRA and hexamethylene bisacetamide have been proven to induce differentiation while simultaneously downregulate the expression of CD44 (114), suggesting that the decrease of CD44 may participate in the therapeutic effect of chemotherapeutic drugs. Furthermore, it has been observed that certain drugs with similar functions may exert their effects through different mechanisms. The regulation of CD44v6 expression by ATRA differs from that of As_2_O_3_ (115). Specifically, in the process of ATRA-induced differentiation, CD44v6 transcript levels are downregulated, while maintain CD44v6 translation levels at their original levels; concurrently, the PI3K/Akt signaling axis is strengthened. Conversely, during As_2_O_3_-initiated differentiation, both transcriptional and translational levels of CD44v6 are strikingly downregulated and the PI3K/Akt pathway is blocked. The other natural compounds and chemotherapeutic drugs have been listed in the **Table 2**.

**Table 2:** The clinical application of CD44 antibodies and their mechanism of action.

Method	drug	type of leukemia	Reference	Mechanism
HA-directed targeting of cancer cells	curcumin liposome modified with HA	AML	(116)	Akt/ERK pathways inhibition and reactivation of tumor suppressor genes
	doxorubicin encapsulated in lipoic acid-crosslinked HA nanoparticles	AML-2, MM	(117)	Enhanced intake concentration
	lipid-substituted polyethylenimine/siRNA complexes	AML	(118)	Reduced adhesion, induction of apoptosis
	poly lactide co-glycolide nanoparticles conjugated with anti-CD44 and encapsulating parthenolide	AML	(119)	Inhibition of NF-kB signaling
	CD44-targeted glutathione-sensitive HA-mercaptopurine 6-Mercaptopurine	AML	(120)	Enhanced growth inhibition, better survival rate
Pharmacological inhibition of CD44	Ampelopsin	APL, CML	(121)	Downregulation of AKT and NF-κB signaling, induction of apoptosis, and inhibition of CD44 expression
	Cyclopamine	AML	(108)	Hedgehog signaling pathway inhibition
	Shikonin	CML	(122)	Upregulation of PTEN and BAX
	nonsteroidal anti-inflammatory drugs	cancer stem-like cells	(123)	Upregulation of LC3-II and downregulation of p62
	Trametinib	AML	(124)	Upregulation of PD-L1
	ATRA/hexamethylene bisacetamide	AML	(114)	Downregulation of cyclin E mRNA, and upregulation of p27 and p21
	Astilbin	Jurkat cells	(125)	Inhibition of TNF-α production and MMP-9 secretion
	Ibrutinib	B-ALL	(126)	PI3K/Akt signaling inhibition
	Sotrastaurin	CLL	(127)	Downregulation of transcriptional genes and BCR-mediated survival pathways
	ATRA, arsenic trioxide	AML	(128)	Downregulation of PI3K/Akt pathway

### 4.3 The exploration of sCD44 and HA oligomers

The sCD44 present in body fluids functions as a competitive inhibitor, counteracting the effect of membrane-bound CD44 and providing valuable insights for CD44-relevant treatments. Previously, sCD44 was primarily utilized for disease monitoring, especially as a prognostic indicator in AML patients. However, recent studies have implicated that sCD44 is involved in the pathogenesis of B-CLL, and propose inhibition of sCD44 as a potential therapeutic strategy (67).

The use of HA oligomers is an alternative approach to inhibit HA-CD44 interaction. It has been demonstrated that monovalent interaction with small oligomers of HA (6–18 saccharide units of HA) effectively reverse the function exerted by high molecular weight HA. Furthermore, this mechanism efficiently inhibits the CXCL12-regulated CXCR4 signaling pathway (129).

### 4.4 Effects of CD44 on the hematopoietic niche

The eradication of residual LSCs following chemotherapy or HSC transplantation is pivotal in preventing leukemia relapse, while the protective role of the hematopoietic niche shields leukemia cells from the cytotoxic effects of chemotherapy drugs. Adhesion is a critical part of normal hematopoiesis and helps LSCs to remain sheltered in the bone marrow microenvironment (130). Its involvement in chemotherapy resistance or relapse after remission poses a significant challenge for treatment. Downregulation of cell-surface adhesion molecules can liberate LSCs from the hematopoietic niche and augment the efficacy of chemotherapeutic interventions. The initiation of HA-CD44 binding is responsible for the involvement of CD44 in the increase of additional adhesion molecules (mostly integrins) and the enhanced adhesion of α4β1 (an integrin) to fibronectin and laminin. An in-depth study of the mechanism of action of adhesion molecules would facilitate the identification of a therapeutic agent that impedes aberrant adhesion between LSCs and the hematopoietic niche, thereby promoting their differentiation, proliferation, and migration outwards. Although LSCs and HSCs share the similar adhesion molecules and signaling pathways in most cases, further research reveal that the adhesion mechanisms of HSCs and LSCs do not completely overlap. Several studies have suggested that BCR-ABL1^+^ LSCs exhibit a greater reliance on selectins and their ligands for homing compared to HSCs (131). Selectin blockade may therefore be a beneficial strategy in the leukemia treatment. CD44 interacts with E- and L- selectin when it is sialo-fucosylated and bears the SleX glycan (132). *In vivo* administration of H90, an activating mAb directed to CD44, has demonstrated the ability to enhance the adhesion of normal HSCs to HA, while concurrently inhibiting the binding of LSCs to HA, and thereby significantly reduce leukemia repopulation (103).

Abnormal glycosylation is a characteristic of cancer cells, and several changes in glycan structure are associated with cancer progression. Selectin-ligand interactions are involved in cancer cell interactions with platelets, leukocytes, and endothelial cells, and as well as promoting tumor cell dissemination through signal transduction pathways. Moreover, selectin-mediated specific interactions between host cells expressing selectins and ligands on tumor cells can block the microvascular system. Inflammatory cytokines produced by cancer cells have been shown to stimulate high expression of E-selectin during early stages of cancer progression (133). CD44v4 is an E-selectin ligand expressed in metastatic breast cancer, playing a crucial role regulating the interaction between cancer cells and endothelial cells through E-selectin. This regulatory mechanism promotes the trans-endothelial migration of cancer cells. In addition, the loose microenvironment and formation of pre-metastatic niches are reported to be critical for the establishment of metastasis and are the reason why circulating cancer cells can colonize distant organs. Selectins and their respective ligands also help maintain the structural integrity of the pre-metastatic niches.

Relevant methods have been explored, encompassing modulation of selectin-ligand interaction, alteration of selectin expression to modify the biosynthesis or cleavage of selectin ligands. Other strategies can also be considered in conjunction with selectin blockade for targeted attack on cancer cells while minimizing interference with normal HSCs, such as immunophenotype transformation, alteration of cell metabolism, epigenetic regulation, and modification of the cellular microenvironment.

Regarding the potential side effects of such interventions, when selectins mediate metastasis through the activation of the inflammatory cascade and participating in shaping the tumor microenvironment, they also help identifying and eliminating tumor cells. The dual functionality of selectins poses challenges for developing effective inhibitors targeting selectin-ligand interactions *in vivo*. On one hand, excessively potent inhibitors may exert detrimental effects on the healing process. On the other hand, weak inhibitors may fail to sufficiently intervene in the pathological processes of serious diseases. Thus, an effective therapeutic agent necessitates a delicate balance in selectin-ligand interactions. Furthermore, structural features of the calcium-dependent carbohydrate recognition domain with a relatively shallow surface pose a challenge to the rational design of selectin inhibitors. Numerous sugar-like or non-carbohydrate and polysaccharide inhibitors have been developed to inhibit selectin-ligand interactions, but despite considerable effort, only a few compounds have shown promising results in clinical trials.

Finally, implementing treatment at different stages of treatment can also significantly influences the outcome, with particular emphasis on targeting LSCs during the initial complete remission (CR) phase when they exhibit greater homogeneity. The limited efficacy of LSC-targeted therapy against a vast population of leukemia cells renders it more advantageous in scenarios characterized by lower tumor burden and reduced tumor heterogeneity.

### 4.5 Precise immunotherapeutic potential of CD44

Immunotherapy refers to the enhancement of therapeutic effects by boosting the anti-tumor immune response and overcoming immune tolerance towards tumor. Moreover, Immunotherapy encompasses not only the manipulation of the tumor microenvironment but also entails regulation of peripheral immune cells. Broadly categorized, immunotherapy can be classified into three types: active immunotherapy, which involves administering low-toxicity tumor vaccines to patients; passive immunotherapy encompassing CAR-T cell therapy or passive delivery of immunosuppressants or anti-tumor cells to patients; and the third type is immunomodulatory site therapy, specifically inhibitors of immune checkpoints or inhibitors of immunomodulatory sites. PD-1 serves as an important immune locus, and inhibition of the PD-1-PD-L1 axis not only blocks immunosuppressive response within tumor cells, but also induces an anti-tumor response in peripheral immune cells (134). CD44 has been proven to promote immune escape in various types of tumor cells, with its high expression being associated with the upregulation of immune escape-related marker proteins (135). In lung cancer, binding of CD44 to Secreted Phosphoprotein1 (SPP1) blocks T-cell proliferation (136). CD44 binds more readily to acetyl HA on antigen-activated T lymphocytes and inflammation-stimulated monocytes (137). Due to its ability to induce immune escape, PD-L1 is targeted by multiple anti-tumor drugs; furthermore, CD44 is positively correlated with the expression of PD-L1 (138). In addition, CD44 may serve as an independent prognostic factor for immune invasion (139). CD44v6 is expressed in a variety of AML cells and is an essential molecule for the proliferation of leukemia cells, while in healthy individuals, HSCs and lymphocytes express minimal levels of CD44v6, which is exclusively found in monocytes. Ex vivo and *in vivo* experiments have demonstrated that CD44v6 CAR-T cells selectively eliminate AML cells while sparing HSCs but targeting monocytes (140).

### 4.6 HA-directed targeting of cancer cells

The development of drugs centered on HA is predicated upon its high affinity for CD44, excellent biocompatibility, and biodegradability (141). HA-mediated targeted therapy can be conceptualized as a therapeutic method that capitalizes on the specific binding between HA and CD44. In this strategy, CD44 serves as the target receptor for interaction while drug-conjugated HA acts as the carrier to transport drugs. By exploiting the specific binding capability, the drug-loaded HA facilitates targeted delivery to cancer cells expressing high levels of CD44. Common applications include HA-modified nanoparticles, HA-linked drug conjugates, and HA-liposomes.

The HA-modified nanocarriers based on HA exhibit non-toxic, non-immunogenic and water-soluble attributed to the inherent characteristics of HA itself. Additionally, nanoparticles possess advantages such as small particle size, high drug loading capacity, controlled drug release, efficient tumor targeting ability, and prolonged *in vivo* circulation time (142). The CD44 receptor specifically binds with high affinity to its natural ligand HA. By considering the chemical structure of the drug itself alone with the specificity of the target site and the affinity towards cell surface molecules, a combination approach is often employed for treating malignancies with overexpression of CD44. The modification of nanoparticles, such as liposomes, carbon nanotubes, and dendritic macromolecules with HA as a surface modification material, can improve the targeting efficiency of nano formulations and relatively prolong the *in vivo* circulation time of drugs (143).

Furthermore, ongoing research is being conducted on the advancement of nanocell and nanogel technologies. The principle of HA nanomicelles is based on the existence of multiple active functional groups in the chemical structure of HA enabling modification of various hydrophobic chains onto or at the end of the HA skeleton to obtain amphiphilic HA derivatives. These derivatives can self-assembled into solution-phase micelles with a core-shell structure in solution, facilitating encapsulation of poorly water-soluble anti-tumor drugs within the hydrophobic core. This approach significantly enhances drug solubility and stability. Nanogels are prepared by cross-linking sulfhydryl groups in HA-SH to form disulfide bonds. Small interfering RNAs (siRNAs) can be physically encapsulated into the nanogels formed by HA-SH under ultrasonic conditions using the reverse water-in-oil emulsion method. In addition, owing to the presence of disulfide bonds inside the nanogel structure, it enables rapid release of encapsulated siRNAs under high GSH concentration conditions (intra-tumor cell GSH concentration) improving gene silencing efficiency.

HA-drug couplers are synthesized as prodrugs through the formation of cleavable chemical bonds between a drug and HA, which improve the solubility and pharmacokinetic properties of the drug, as well as facilitating its cellular uptake by CD44 receptor mediated endocytosis. The interaction of CD44 and HA can be exploited as a promising therapeutic target for targeted drug delivery due to its high affinity. The typical composition of antibody-drug conjugates (ADCs) include three main components: (1) a mAb with the function of accurately selective location; (2) an effective cytotoxic small molecule; and (3) a linker responsible for the connection between two substances (mainly liposomes, hydrogels, and nanoparticles) which has been proven to promote the therapeutic effects under the same amount of the chemotherapeutic drugs and minimize the systemic toxicities to some extent. The results may be related to the mechanism by which the high affinity between HA-decorated liposomes and specific receptors leads to fully internalization of drugs. Besides, compared with other normal ADCs, HA can be exploited to entrap and transport drugs to CD44-expressing tumors without the requirement of chemical conjugation due to its enormous hydrodynamic domain. The drug-antibody ratio, or the amount of drug molecules attached to a single ADC, is an important indicator for assessing the function and toxicity risk of ADC drugs. High drug loads affect toxicities and pharmacokinetics while low drug loads may diminish potency. In addition, the systemic stability of ADCs after administration encompasses metabolic stability and integrity, which are another important factor determining their efficacy. Consequently, targeted research has focused on stabilizing ADCs through conjugate site selection and joint modification. The selection of attachment sites with larger steric hindrance has been proven to be an effective approach for providing the desired spatial shielding for antibodies. Conversely, introducing proximal steric hindrance around the splice or instability sites of the joint can also effectively improve stability. Other novel ADCs include immunostimulants, such as toll-like receptor agonists, STING agonists or chemokines, which better attract immune cells to tumor cells and further enhance immune cell activities. However, it should be noted that the distribution of cytotoxic drugs is mostly determined by the level of target antigen expression, which can occasionally result in unexpectedly severe “targeted, detumorable” toxicity that is not strictly proportional to the payload. Furthermore, similar to other pharmaceutical agents, the active cytotoxic components carried by ADCs undergo metabolic changes. Thus, the risk of drug interaction and other allergies should be considered, and necessary measures should be taken to eliminate relevant side effects.

## 5 Conclusion

CD44, a significant biomarker, serves as a platform for intercellular communication and intracellular signaling pathways that play a crucial role in regulating cellular behavior. It is also an established component of the LSC niche, which represents a promising target for anti-cancer therapy. Several strategies have been applied to inhibit CD44 for cancer therapy, including HA nanoparticles, small-molecule inhibitors, and anti-CD44 mAbs. These approaches are showing promising results in preclinical research. Among the various mechanisms reversed by anti-CD44 antibody, the inhibition of LSC differentiation has attracted more interests due to its crucial role in mediating HSC engraftment into the bone marrow niche. As LSCs share most of the same mechanisms as HSCs, finding ways to selectively target LSCs without disrupting HSC function has become a pressing issue. In conclusion, targeting CD44 represents a promising therapeutic strategy for controlling the progression of AML. Therefore, it is imperative to comprehensively elucidate the mechanisms underlying HA-CD44 interaction and signaling in order to identify rational treatments that specifically target CD44 and enhance therapeutic efficacy.

## Author contributions

SW: Writing – original draft. YT: Writing – original draft. FL: Writing – review & editing. YH: Writing – review & editing. SZ: Conceptualization, Supervision, Writing – review & editing. XL: Visualization, Writing – review & editing.
